# Immunometabolic integration in therapeutic strategies for managing MASLD

**DOI:** 10.3389/fphar.2025.1693753

**Published:** 2025-11-10

**Authors:** Sergio González-Serrano, Alina-Iuliana Onoiu, Jordi Camps, Jorge Joven

**Affiliations:** 1 Unitat de Recerca Biomèdica, Hospital Universitari Sant Joan, Institut d’Investigació Sanitària Pere Virgili, Universitat Rovira i Virgili, Reus, Spain; 2 Department of Medicine and Surgery, Faculty of Medicine, Universitat Rovira i Virgili, Reus, Spain; 3 The Campus of International Excellence Southern Catalonia, Tarragona, Spain

**Keywords:** genetics, GLP-1, inflammation, lipid metabolism, MASH, mitochondrial dysfunction, obesity, pharmacotherapies

## Abstract

Metabolic dysfunction-associated steatotic liver disease (MASLD) is clinically complex. Management approaches have focused on addressing the traits of metabolic syndrome and promoting weight loss. However, current treatment options are inadequate, leaving key disease-driving factors unaddressed. Evidence suggests that immune dysregulation determines disease trajectory. Metabolic pathways shape the immune landscape, and the immune system influences metabolic homeostasis. Developing therapies that integrate metabolic correction with immune restoration is essential. We review current available strategies and discuss areas where further research is needed to design drugs and therapeutic combinations that mitigate the complex metabolic and inflammatory interactions driving obesity-associated chronic liver disease. The immunomodulatory effects of obesity-focused interventions remain poorly understood. Bariatric surgery and incretin-based therapies can reduce body fat while reprogramming the hepatic immune environment. Metabolic modulators can reduce lipotoxicity, suppress harmful cytokine networks, and promote reparative immune responses. Other strategies include blocking danger-signaling pathways, modulating chemokine axes, and using cellular therapies. The goal is to interrupt pro-inflammatory amplification cascades and preserve reparative immune cell populations, redefining therapeutic possibilities for liver diseases. Despite advancements in the field, uncertainties still exist regarding the immunometabolic integration. Ongoing clinical trials and the recent approval of two drugs for treating this condition will provide valuable real-world insights in the future about the long-term safety and effectiveness of potentially more accurate treatment approaches. Moreover, causal and clinical biomarkers are being investigated to enhance the diagnosis and management of the significant challenges associated with MASLD-related cirrhosis. Prioritizing and initiating treatment earlier are key factors for achieving successful outcomes.

## Introduction

In 1980, specific histologic features identified a then-new and rare chronic liver condition associated with obesity and diabetes mellitus, termed nonalcoholic steatohepatitis, as patients consistently reported no history of chronic alcohol abuse (Ludwig et al., 1980). This condition represented the progressive form of nonalcoholic fatty liver disease. Concurrent with rising obesity prevalence over subsequent decades, metabolic dysfunction emerged as a central driver of progression from hepatic fat accumulation to inflammation and fibrosis. Contemporary nomenclature reflects this mechanistic understanding: metabolic dysfunction-associated steatotic liver disease (MASLD) and its inflammatory stage, metabolic dysfunction-associated steatohepatitis (MASH), have replaced earlier terms (Kanwal et al., 2024). MASLD has evolved from a rare entity to a multisystem disease affecting one-quarter of the global population and constituting a substantial global health burden, with implications extending to extrahepatic complications (Estes et al., 2018; Younossi et al., 2021; Tacke et al., 2024).

MASLD represents a major obesity-related complication characterized by genetic predisposition, disrupted gut-liver and adipose-liver axis signaling, and persistent immune dysfunction (Anstee et al., 2019; Loomba et al., 2021). While promising metabolism-based drugs targeting MASLD are emerging, immune mechanisms linking metabolic injury to inflammation and fibrosis require further investigation (Peiseler et al., 2022). The liver functions as an immunological sentinel. The immune dysregulation caused by obesity primarily affects the adipose tissue and extends to the liver and other metabolic organs (Hotamisligil, 2006). Current models suggest that transition to a pro-inflammatory hepatic environment begins with stress sensing by resident immune cells, followed by recruitment of pro-inflammatory myeloid populations and subsequent lymphocyte engagement (Mladenić et al., 2024). However, conceptualizing the immune system as a unidirectional driver of tissue injury oversimplifies the pathophysiology. Immune cells can orchestrate tissue repair and restore hepatic homeostasis (Zhao et al., 2022). This dual function necessitates a comprehensive characterization of immune profile changes and nuanced risk stratification across clinical contexts. The emerging immunometabolic paradigm challenges the traditional perception of MASLD as a purely metabolic disease with secondary immune dysregulation. Instead, it supports an integrated framework that recognizes metabolic pathways shape immune functions, while immune responses determine metabolic homeostasis. This complexity provides the foundation for therapeutic approaches that synchronize metabolic correction with immune restoration in current and future drugs.

## Weight loss-related strategies

The association between obesity and MASLD suggests that effective anti-obesity treatments can contribute substantially to disease remission (Brunner et al., 2019; Polyzos et al., 2019). However, these approaches achieve therapeutic benefits not merely through weight reduction, but by simultaneously remodeling the hepatic immune microenvironment. Weight loss through lifestyle modifications remains the cornerstone of MASLD management. Sustained reduction of 7%–10% body weight improves steatosis and lobular inflammation, whereas losses exceeding 10% are required to reverse fibrosis (Vilar-Gomez et al., 2015). Bariatric surgery extends these effects through greater and sustained weight loss, improving insulin sensitivity and glycemic control, downregulating *de novo* lipogenesis (DNL), and upregulating lipid-oxidation genes (Lee et al., 2019; Lassailly et al., 2020; Verrastro et al., 2023; Liu et al., 2025). However, some patients may not experience halted MASLD progression or reversal of advanced fibrosis despite substantial weight loss (Pais et al., 2022).

The efficacy of bariatric surgery on MASLD transcends energy balance through direct immunomodulatory mechanisms. This procedure reduces systemic inflammatory markers (CRP, LBP, HGF, IL-6, and TNFα) while promoting gut microbiome shifts that increase hepatic natural killer T cells and reduce pro-inflammatory macrophages (Shera et al., 2024). Evidence from murine sleeve-gastrectomy models indicates that TREM2^+^ macrophages emerge as key mediators of post-surgical hepatic improvements (Fredrickson et al., 2024). These cells contribute to the clearance of cellular debris, the attenuation of local inflammation, and the remission of fibrosis. In the liver, TREM2^+^ macrophages localize to hepatic crown-like structures that encircle lipid-laden apoptotic hepatocytes, promoting their efferocytosis. Conversely, prolonged hypernutrition activates ADAM17 metalloproteinase through TNFα and IL-1β signaling, leading to TREM2 cleavage and impaired macrophage efferocytosis, thereby promoting disease progression (Daemen et al., 2021; Wang et al., 2023; Ganguly et al., 2024; Zhou et al., 2024). Although preliminary clinical observations align with these findings, validation in larger cohorts remains necessary.

**FIGURE 1:**
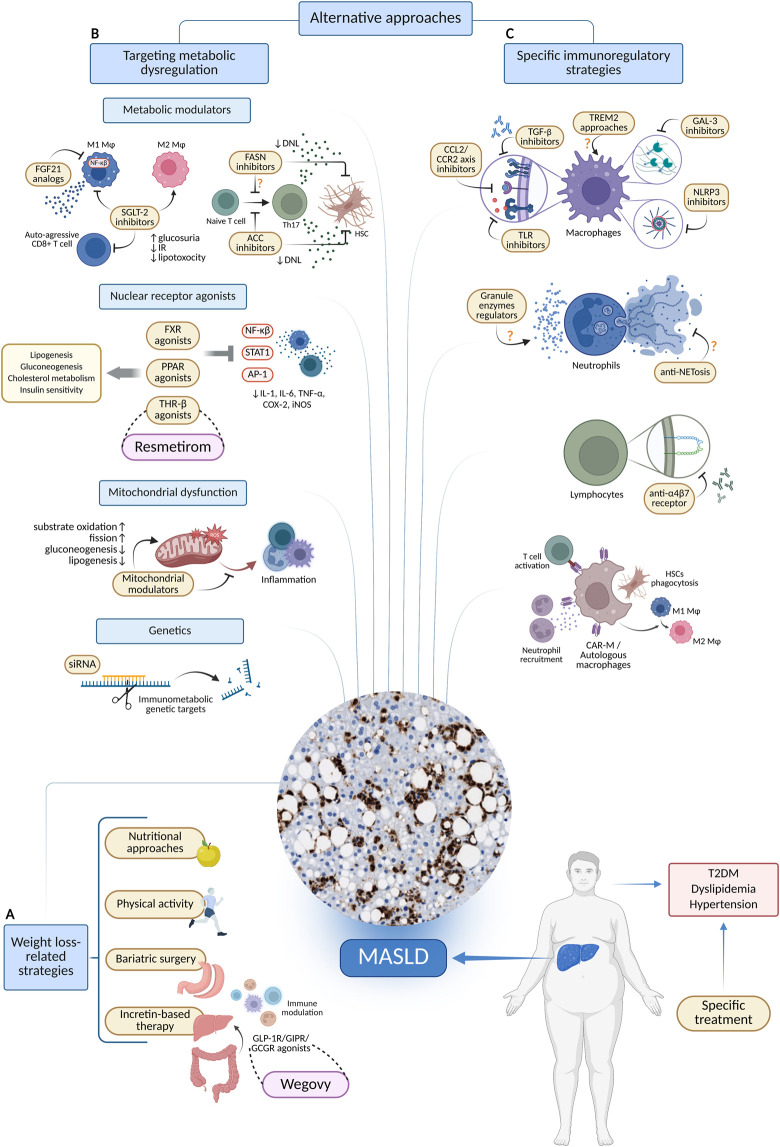
Therapeutic approaches targeting metabolic and immune alterations in MASLD. **(A)** Strategies associated with obesity. **(B)** Therapies focused on metabolism. **(C)** Specific strategies for immune modulation. Created in BioRender. Joven, J. (2025) https://BioRender.com/d9mzal6. Abbreviations: ACC, acetyl-CoA carboxylase; AP-1, activator protein 1; CAR-M, chimeric antigen receptor modified macrophage; CCL2, C-C motif chemokine ligand 2; CCR2, C-C chemokine receptor 2; COX-2, cyclooxygenase 2; DNL, *de novo* lipogenesis; FASN, fatty acid synthase; FGF21, fibroblast growth factor 21; GAL3, galectin 3; GCGR, glucagon receptor; GIPR, glucose dependent insulinotropic polypeptide receptor; GLP-1, glucagon like peptide 1; HSC, hepatic stellate cell; IL-1, interleukin 1; IL-6, interleukin six; iNOS, inducible nitric oxide synthase; MASLD, metabolic dysfunction-associated steatotic liver disease; NET, neutrophil extracellular trap; NF-κB, nuclear factor of the κ chain in B cells; NLRP3, nucleotide binding domain, leucine-rich repeat protein 3; SGLT-2, sodium glucose cotransporter 2; siRNA, small interfering RNA; STAT1, signal transducer and activator of transcription 1; T2DM, type 2 diabetes mellitus; TGF-β, transforming growth factor β; Th17, T helper 17 cells; THR-β, thyroid hormone receptor β; TLR, toll like receptor; TNFα, tumor necrosis factor alpha; TREM2, triggering receptor expressed on myeloid cells 2.

Incretin-based therapies represent a paradigm shift from weight loss-dependent to dual metabolic-immune targeting strategies. In obesity, gut barrier dysfunction and dysbiosis enable microbial products to reach the liver via the portal vein, activating resident immune cells and sustaining inflammation and progression of MASLD (Tilg et al., 2021). In contrast, incretins secreted in the gut, such as glucagon-like peptide-1 (GLP-1) and glucose-dependent insulinotropic polypeptide (GIP), represent beneficial signals that can be therapeutically harnessed. GLP-1 receptor (GLP-1R) agonists are the most effective incretin-based therapies (Newsome and Ambery, 2023). These agents facilitate weight loss and glycemic control by enhancing satiety and stimulating glucose-dependent insulin secretion (Drucker, 2022; Newsome and Ambery, 2023). Beyond metabolic effects, their capacity to suppress inflammation across multiple tissues positions them as immunomodulatory agents relevant to MASLD (He et al., 2025). Clinically, liraglutide and semaglutide increase rates of histological resolution *versus* placebo (Armstrong et al., 2016; Sanyal et al., 2025). In phase III clinical trials (ESSENCE), semaglutide also promoted fibrosis remission and improved noninvasive fibrosis markers. Interim analysis of histological results resulted in approval by the FDA in 2025 as wegovy (US Food and Drug Administration, 2025). Multi-receptor incretin agonists, currently in phase II or early phase III trials, aim to enhance therapeutic efficacy by incorporating GIP or glucagon receptor activation. Examples include dual GIP/GLP-1 receptor agonists (tirzepatide), dual GLP-1/glucagon receptor agonists (survodutide, pemvidutide, cotadutide), and triple GLP-1/GIP/glucagon receptor agonists (retatrutide). However, their specific immunomodulatory mechanisms remain to be fully characterized (Loomba et al., 2024b; Sanyal et al., 2024; Shankar et al., 2024; Harrison et al., 2025) (Figure 1A).

## Metabolic dysregulation and immunomodulation

In MASLD, macrophages, T cells, and other immune populations undergo metabolic reprogramming (Ma et al., 2025). This immunometabolic dimension could inform therapeutic strategies that target core metabolic pathways independent of weight loss while simultaneously modulating immune functions (Figure 1B). Nuclear receptor agonists represent promising therapeutic targets, as they activate transcription factors that regulate both metabolism and immunity. Among them, resmetirom, an oral liver-directed **thyroid hormone receptor-β (THR-β)** selective agonist, exemplifies this approach. Following the demonstration of increased rates of MASH resolution and improvement in fibrosis in the MAESTRO-NASH phase 3 trial, resmetirom received accelerated FDA approval as the first pharmacological therapy for MASH (Harrison et al., 2024; Keam, 2024). Mechanistically, resmetirom acts on the expression of key enzymes and modulates hepatic lipid metabolism by enhancing triglyceride breakdown and reducing DNL. Several data suggest a significant role in mitochondrial biogenesis, improving hepatic fatty acid oxidation and attenuating lipotoxicity-driven inflammation (Petta et al., 2024). The **farnesoid X receptor (FXR)** illustrates the complexity of nuclear receptor targeting. FXR functions as a bile acid sensor regulating cholesterol and bile acid homeostasis, gluconeogenesis, and lipogenesis (Fiorucci et al., 2022). It is expressed in monocytes, macrophages, dendritic cells, natural killer cells, and natural killer T cells, where its activation exerts anti-inflammatory effects by inhibiting NF-κB (Fiorucci et al., 2022). However, obeticholic acid, the most prominent FXR agonist, has shown limited success in MASH resolution and raised safety concerns (Sanyal et al., 2023). **Peroxisome proliferator-activated receptors (PPARs)** represent archetypal nodes of immunometabolism. PPARs function as fatty acid sensors regulating lipid metabolism, insulin sensitivity, inflammation, and fibrogenesis (Francque et al., 2021). All PPAR isotypes (PPARα, PPARγ and PPARβ/δ) are expressed in immune cells, including macrophages, neutrophils, dendritic cells, and T cells, where they modulate anti-inflammatory responses (Christofides et al., 2021). They attenuate macrophage and neutrophil activation by limiting leukotriene B4 signaling, suppressing NF-κβ, AP-1 and STAT1 pathways, and downregulating pro-inflammatory mediators such as IL-1, IL-6, TNF-α, COX-2, and iNOS (Qiu et al., 2023; Staels et al., 2023). PPARγ also favors anti-inflammatory M2 macrophage polarization via glutamine metabolism. These effects provide a robust mechanistic rationale for indicating PPARs in MASLD. Lanifibranor, a pan-PPAR agonist, has demonstrated significant reductions in hepatic steatosis and improvements in tissue insulin resistance in a phase 2 trial (Barb et al., 2025). This agent reduces hepatic macrophage accumulation, downregulates their pro-inflammatory gene programs, and upregulates lipid-handling gene expression (Lefere et al., 2020).

Beyond nuclear receptors, other metabolic modulators demonstrate how targeting specific metabolic pathways can directly reprogram immune cell function. A notable example is the **sodium-glucose cotransporter 2 (SGLT-2)**, a membrane protein that reabsorbs most of the filtered glucose in the early proximal tubules of the kidney. Pharmacologic SGLT-2 inhibitors promote glycosuria, reduce insulin resistance and lipotoxicity, and create sustained caloric deficits, leading to decreased body weight and concomitant hepatic improvements in MASLD (Ong Lopez and Pajimna, 2024). These metabolic shifts are coupled to direct immunoregulatory actions. In murine models, empagliflozin ameliorates MASH by directly suppressing auto-aggressive CXCR6^+^ CD8^+^ T cell activation and infiltration through ketogenesis-dependent mechanisms (Liu et al., 2024). It also enhances autophagy in hepatic macrophages via AMPK/mTOR signaling, suppressing the IL-17/IL-23 axis (Meng et al., 2021). Similarly, dapagliflozin and canagliflozin inhibit M1 macrophage glycolysis and promote reprogramming toward oxidative metabolism consistent with M2 polarization in the murine liver (Lin et al., 2024). However, clinical evidence supporting these immunomodulatory mechanisms remains limited. **Fibroblast growth factor 21 (FGF21)** is a liver-derived endogenous hormone whose pharmacologic analogs exhibit therapeutic promise in obesity, type 2 diabetes, and MASLD by regulating lipid metabolism and enhancing insulin sensitivity (Jin et al., 2023). Beyond metabolic effects, FGF21 suppresses macrophage expression of pro-inflammatory cytokines, including TNFα, IL-6, IL-1β, and IFN-γ, by promoting NRF2 nuclear translocation and inhibiting NF-κB signaling (Yu et al., 2016). Consistently, FGF21 analog administration in obese nonhuman primates with MASLD reduced hepatic neutrophil and macrophage infiltration (Cui et al., 2020). In phase IIb studies, pegozafermin improved fibrosis, whereas efruxifermin achieved both fibrosis and MASH resolution (Harrison et al., 2023; Loomba et al., 2023).

Targeting ***de novo* lipogenesis** also provides evidence for immunometabolic integration. While fibrosis is the principal predictor of MASLD-related mortality, hepatic steatosis initiates the inflammatory cascade leading to disease progression. Targeting rate-limiting enzymes in hepatic DNL pathways represents a rational therapeutic approach with additional benefits stemming from immune cell modulation (Castro Cabezas and de Jong, 2025). **Acetyl-CoA carboxylase (ACC)** enables T cell membrane phospholipid synthesis and Th17 differentiation, with pharmacological inhibition reducing IL-17 production and alleviating MASH (Ross et al., 2020). Downstream of ACC, **fatty acid synthase (FASN)** is a critical immunometabolic node for Th17 differentiation, but this function remains to be determined in humans (Young et al., 2017). The oral FASN inhibitor denifanstat achieved clinically significant MASH and fibrosis resolution in a phase IIb trial (Loomba et al., 2024a). Certain metabolic interventions target hepatic stellate cell (HSC) transdifferentiation into myofibroblasts, a process dependent on metabolic reprogramming involving enhanced glycolysis, glutaminolysis, and DNL (Martínez García de la Torre et al., 2025). Preclinical models demonstrate that ACC and FASN inhibitors reduce HSC-driven matrix deposition and fibrosis (Ross et al., 2020; O’Farrell et al., 2022). Enhancing PPAR-γ activation also induces HSC quiescence and reduces fibrogenic capacity (Lefere et al., 2020).

## Cutting-edge mitochondrial and genetic approaches

Mitochondrial dysfunction represents a fundamental immunometabolic target in MASLD. The resulting oxidative stress triggers and perpetuates inflammatory responses, establishing mitochondria as promising immunoregulatory targets (Hernández-Aguilera et al., 2013). HU6, metabolized in the liver to the mitochondrial uncoupler 2,4-dinitrophenol, acts as a metabolic accelerator promoting lipid and substrate oxidation. In MASLD patients with elevated BMI, it demonstrated dose-dependent reductions in hepatic fat content relative to placebo (Noureddin et al., 2023). MSDC-0602K, a mitochondrial pyruvate carrier inhibitor, failed to meet histological endpoints in phase II clinical trials in patients with MASH (Harrison et al., 2020). However, the highest doses demonstrated reductions in fasting glucose, insulin, ALT, and AST levels. This approach, as a therapeutic strategy, remains experimental. Metformin represents another plausible MASLD therapy targeting mitochondrial alterations. It activates AMPK, facilitating mitochondrial fission and mitophagy, suppressing hepatic gluconeogenesis and lipogenesis, and enhancing fatty acid oxidation (Perazza et al., 2024). In experimental models, the combination of metformin and genistein exerts immunoregulatory effects by polarizing macrophages toward the anti-inflammatory M2 phenotype (Zamani-Garmsiri et al., 2021).

Emerging evidence demonstrates that specific genetic variants orchestrate the complex interplay between metabolic dysfunction and immune cell dynamics in MASLD (Sookoian et al., 2020). Metabolism-related genes proposed as MASLD progression biomarkers, including HPRT1, GPD1, and GCK, correlate with hepatic immune cell infiltration (Xie et al., 2025). Additionally, a Mendelian randomization study demonstrated the association of eight genetically determined immunophenotypes with MASLD risk (Ye et al., 2024). These insights are guiding the development of targeted genetic therapies that address the fundamental genetic determinants of immunometabolic dysfunction. Current investigational approaches for MASLD use small-interfering RNAs to mimic the protective loss-of-function of HSD17B13 variant with agents such as GSK4532990 and ALN-HSD, with early signs of reduction of liver fat and stiffness in clinical trials (Noureddin, 2024).

## Challenges in specific immunoregulatory strategies

MASLD evolves within a highly interactive immune niche. Therefore, conventional agents providing broad immunosuppression are unsuitable (Tacke et al., 2023). Precision immunomodulation specifically targets immune cell signaling, trafficking, and effector functions. Currently, clinical trial failures are learning opportunities rather than dead ends (Figure 1C; Table 1).

**TABLE 1:** Ongoing strategies for MASLD considering immunoregulatory effects.

Pharmacological agent	Mechanism of action	Immunological outcomes	Evidence type
Liraglutide (Armstrong et al., 2016; Li et al., 2019)	GLP-1 receptor agonist	Evidence of Kupffer cell M2 polarization	Preclinical
Cotadutide (Boland et al., 2020; Shankar et al., 2024)	Dual GLP-1/glucagon receptor agonist	Reduction of hepatic ALT and AST in humans. Reduced hepatic immune cell infiltration and inflammatory markers in murine models. Discontinued after phase II trials for strategic portfolio considerations	Clinical/Preclinical
Resmetirom (Harrison et al., 2024)	THR-β agonist	Attenuated lipotoxicity-driven inflammation by modulating lipid metabolism	Clinical
Lanifibranor (Lefere et al., 2020)	pan-PPAR agonist	Reduced hepatic monocyte-derived macrophage infiltration and activation	Preclinical
Empagliflozin (Meng et al., 2021; Liu et al., 2024)	SGLT-2 inhibitors	Suppressed auto-aggressive CD8^+^ T cell subsets activation by modulating ketogenesis. Enhanced hepatic macrophage autophagy via AMPK activation and mTOR inhibition, suppressing IL-17/IL-23 axis	Preclinical
Dapagliflozin (Lin et al., 2024; Liu et al., 2024)		Reduced CD8^+^ T cell infiltration in human livers. Induced metabolic reprogramming of hepatic murine macrophages and M2 polarization	Clinical/Preclinical
PF-05221304 (Ross et al., 2020)	ACC inhibitor	Inhibition of CD4^+^ T cells polarization into Th17 cells	Preclinical
Metformin and genistein (Zamani-Garmsiri et al., 2021)	AMPK activators	Reduced hepatic macrophage infiltration and induced M2 polarization	Preclinical
Belapectin (Chalasani et al., 2020)	Galectin-3 inhibitor	Despite promising preclinical data, no clinical efficacy has been proven for fibrosis or inflammation	Clinical
Cenicriviroc (Anstee et al., 2024)	Dual CCR2/CCR5 antagonist	Diminished monocyte recruitment. Discontinued after phase III trials showed no antifibrotic efficacy	Clinical
CAR-Ms (Dai et al., 2024)	Macrophages expressing an anti-uPAR CAR	Phagocytose uPAR^+^ HSCs and stimulate T-cell antifibrotic responses, neutrophil recruitment, NK cell activation, and macrophage anti-inflammatory functions	Preclinical
Autologous macrophages (Brennan et al., 2025)	Macrophage therapy targeting fibrosis	No statistically significant differences in phase II trial, but a proven safety profile	Clinical

### Macrophage-targeted interventions

Hepatic macrophages represent primary targets for cell subset-specific approaches, functioning as early sentinels of metabolic stress that rapidly polarize toward pro-inflammatory states. Investigational interventions at this stage focus on upstream pattern-recognition and danger-sensing pathways, specifically inhibiting TLR signaling or blocking NLRP3 inflammasome activation (Yu et al., 2022; 2024). Macrophages represent a significant source of the pro-fibrotic mediator galectin-3. Galectin-3-deficient mice demonstrated protection against fibrosis through inhibition of TGF-β-mediated myofibroblast activation (Subramanian et al., 2022; Sotoudeheian, 2024). However, the clinical evaluation of the galectin-3 inhibitor belapectin in patients with MASH, cirrhosis, and portal hypertension failed to demonstrate significant fibrosis reduction (Chalasani et al., 2020). Moreover, TGF-β neutralization with antibodies such as fresolimumab and metelimumab has been investigated in other diseases but discontinued due to unacceptable risks, including immunosuppression and tumorigenesis (Györfi et al., 2018).

Therapeutic efforts targeting the CCL2-CCR2 chemokine axis, including the dual CCR2/CCR5 antagonist cenicriviroc, aimed to inhibit monocyte recruitment to the liver. Despite initial promise, cenicriviroc did not demonstrate sustained antifibrotic efficacy in phase III trials and was discontinued as a monotherapy for MASH (Anstee et al., 2024). Given the role of chemokines in orchestrating hepatic immune cell trafficking, modulation of these axes remains of therapeutic interest. However, broad suppression of monocyte infiltration risks collateral effects on reparative subsets, including TREM2^+^ macrophages (Hendrikx et al., 2022). To harness the beneficial effects of TREM2^+^ macrophages, several strategies have been proposed. These include direct TREM2 agonism, gene therapy to enhance TREM2 expression, and modulation of gut permeability and dysbiosis to improve TREM2 activation in hepatic macrophages (Shi et al., 2025).

### Neutrophil and T cell modulation

Neutrophils exemplify the complexity of immune targeting in MASLD. They contribute to hepatic injury through granule enzyme secretion and neutrophil extracellular trap (NET) formation (Chen et al., 2021). Although pharmacological modulation of neutrophil-derived factors and tissue infiltration control is being explored in various conditions, MASLD-specific efficacy remains undetermined (Hwang et al., 2021). Anti-NETosis strategies may hold promise for curbing inflammation, fibrosis, and HCC progression (van der Windt et al., 2018). In contrast, neutrophils perform reparative functions in MASLD. They promote macrophage polarization toward pro-resolving states via ROS and deliver miRNA-223 to suppress inflammatory programs in hepatocytes while restraining fibrogenic signaling in HSCs (Calvente et al., 2019; Yang et al., 2019; He et al., 2021; Wang et al., 2021). However, therapeutic strategies designed to enhance neutrophil-mediated repair remain undeveloped.

T cell-targeted approaches have been evaluated in preclinical models. For example, monoclonal antibodies targeting the integrin α4β7 receptor have demonstrated efficacy in preventing CD4^+^ T cells migration to both the intestinal tract and the liver, resulting in a reduction of hepatic inflammation and fibrotic progression (Rai et al., 2020). Clinical efficacy and safety of these strategies remain to be established in human cohorts.

### Immune-mediated fibrosis remission

Hepatic fibrosis demonstrates unique potential for reversibility compared to fibrosis in other organs (Sun and Kisseleva, 2015; Pei et al., 2023). Accumulating evidence indicates that fibrosis remission is, to a significant extent, an immune-mediated process involving macrophages, neutrophils, natural killer cells, and T cells (Tacke et al., 2023). Therefore, immune cells have been targeted for this purpose. In preclinical models, chimeric antigen receptor-modified macrophages (CAR-Ms) targeting the urokinase plasminogen activator receptor (uPAR) have shown significant efficacy in reducing liver fibrosis (Dai et al., 2024). CAR-Ms exert antifibrotic effects by phagocytosing activated HSCs, presenting antigens to CD4^+^ and CD8^+^ T cells, and promoting neutrophil recruitment and macrophage polarization toward anti-inflammatory phenotypes. However, CAR-Ms therapy remains experimental, with clinical applicability yet to be established. Furthermore, clinical studies of autologous macrophage infusion have demonstrated improvements in fibrosis (Moroni et al., 2019). Although a subsequent phase II randomized trial in compensated cirrhosis did not replicate those results, the treated cohort experienced few liver-related events and no deaths, underscoring the need for further evaluation in larger trials (Brennan et al., 2025).

## Perspectives

The complex nature of MASLD necessitates therapeutic strategies that acknowledge it as an immunometabolic disorder. Ongoing data are transforming drug development, shifting the focus from treatments that rely solely on weight loss to approaches that target the interaction between metabolism and the immune system. Therapies that concentrate exclusively on metabolic pathways often provide anti-inflammatory and antifibrotic benefits by reducing metabolic damage. However, the lasting reversal of fibrosis may be limited. Strategies targeting immune cell functions must strike a balance between suppressing inflammation and preserving protective mechanisms. Over-suppression can compromise the immune response and liver clearance functions, while unchecked immune activation can lead to tissue damage and progressive fibrosis. Some cellular therapies, such as CAR-macrophage treatments and autologous macrophage infusions, illustrate the use of intentional immune modulation to address liver diseases. These methods can reprogram immune cells to reverse disease progression rather than exacerbating it. It is also important to consider the combination of synergistic mechanisms, which improves tolerability and reduces the likelihood of developing therapeutic resistance. The heterogeneity of MASLD necessitates tailored therapeutic strategies instead of generic solutions. Precision medicine approaches, informed by new insights into genetic variants related to metabolism and immune interactions, have the potential to enhance patient stratification and tailor treatments to individual disease mechanisms. The clinical implementation of these approaches relies on the development of biomarkers that can simultaneously capture both metabolic dysfunction and immune activation states. These biomarkers are essential for optimizing trial design and monitoring therapeutic responses.

The future of treating MASLD lies not in choosing between metabolic and immune interventions, but in integrating both systems to halt disease progression and restore liver balance. A comprehensive understanding of immune dynamics in liver disease is essential for developing next-generation therapies that effectively combine metabolic and immunological targets. This potential integration represents a promising pathway to transform MASLD from a progressively worsening condition into one that is treatable and reversible.

## Glossary

ACC: acetyl-CoA carboxylase
ADAM17: A disintegrin and metalloproteinase 17
ALT: alanine aminotransferase
AMPK: adenosine monophosphate-activated protein kinase
AP-1: activator protein 1
AST: aspartate aminotransferase
BMI: body mass index
CAR-M: chimeric antigen receptor macrophage
CCL2: C-C motif chemokine ligand 2
CCR2: C-C chemokine receptor type 2
CCR5: C-C chemokine receptor type 5
CD4: cluster of differentiation 4
CD8: cluster of differentiation 8
COX-2: cyclooxygenase-2
CRP: C-reactive protein
CXCR6: C-X-C chemokine receptor type 6
DNL: *de novo* lipogenesis
FASN: fatty acid synthase
FDA: food and drug administration
FGF21: fibroblast growth factor 21
FXR: farnesoid X receptor
GCK: glucokinase
GIP: glucose dependent insulinotropic polypeptide
GLP-1: glucagon like peptide 1
GLP-1R: glucagon like peptide 3 receptor
GPD1: glycerol 3 phosphate dehydrogenase 1
HCC: hepatocellular carcinoma
HGF: hepatocyte growth factor
HPRT1: hypoxanthine phosphoribosyltransferase 1
HSC: hepatic stellate cells
HSD17B13: hydroxysteroid 17-beta dehydrogenase 13
IFN-γ: interferon gamma
IL-1: interleukin 1
IL-1β: interleukin 1 beta
IL-6: interleukin 6
IL-17: interleukin 17
IL-23: interleukin 23
iNOS: inducible nitric oxide synthase
LBP: lipopolysaccharide binding protein
MASH: metabolic dysfunction-associated steatohepatitis
MASLD: metabolic dysfunction-associated steatotic liver disease miRNA-223, microRNA-223
mTOR: mechanistic target of rapamycin
NET: neutrophil extracellular traps
NF-κB: nuclear factor of the κ chain in B cells
NLRP3: nucleotide binding domain, leucine-rich repeat protein 3
NRF2: nuclear factor erythroid 2 related factor 2
PPARα: peroxisome proliferator activated receptor alpha
PPARβ/δ: peroxisome proliferator activated receptor beta/delta
PPARγ: peroxisome proliferator activated receptor gamma
RNA: ribonucleic acid
ROS: reactive oxygen species
SGLT-2: sodium glucose cotransporter 2
STAT1: signal transducer and activator of transcription 1
TGF-β: transforming growth factor beta
Th17: T helper 17 cells
THR-β: thyroid hormone receptor beta
TLR: toll like receptor
TNFα: tumor necrosis factor alpha
TREM2: triggering receptor expressed on myeloid cells 2
uPAR: urokinase type plasminogen activator receptor.
